# Solid Oxygen-Purifying (SOP) Filters: A Self-Disinfecting Filters to Inactivate Aerosolized Viruses

**DOI:** 10.3390/ijerph17217858

**Published:** 2020-10-27

**Authors:** Michael Versoza, Jaeseok Heo, Sangwon Ko, Minjeong Kim, Duckshin Park

**Affiliations:** 1 Transportation Environmental Research Team, Korea Railroad Research Institute, Uiwang City 16105, Korea; mikeverz23@krri.re.kr (M.V.); jsheo1005@krri.re.kr (J.H.); sko@krri.re.kr (S.K.); mjkim88@krri.re.kr (M.K.); 2 Railway System Engineering, University of Science and Technology, Daejeon City 34113, Korea

**Keywords:** self-cleaning filters, viral aerosol inactivation, solid oxygen-purifying filters, HVAC filter application, viral protection, face mask application

## Abstract

Normal heating, ventilation, and air conditioning (HVAC) systems typically use high-efficiency particulate air (HEPA) filters, which can filter dust, various pollutants, and even bacteria and viruses from indoor air. However, since HEPA filters cannot not clean themselves and due to the nature of these microbes which can survive for long periods of time, changing these filters improperly could transmit pathogenic bacteria or viruses, and could even lead to new infections. This study indicated that these manufactured Solid Oxygen-purifying (SOP) filters have the potential to self-disinfect, filter, and inactivate aerosolized viruses. MS2 bacteriophage was used as a model virus in two different experiments. The first experiment involved aerosolization of the virus, while the second were a higher viral load using a soaking method. The SOP filters inactivated up to 99.8% of the virus particles in both experiments, provided that the density of the SOP filter was high. Thus, SOP filters could self-clean, which led to protection against airborne and aerosolized viruses by inactivating them on contact. Furthermore, SOP filters could be potentially use or addition in HVAC systems and face masks to prevent the transmission of airborne and aerosolized viruses.

## 1. Introduction

Pathogenic viruses can be harmful to public health. Aerosol transmission, which is one way to spread pathogens, refers to transmission via particles with diameters smaller than 5 μm, while droplet transmission refers to transmission via particles with diameters of ~10 μm [1,2,3]. Viruses are mainly spread through coughing or sneezing, which results in the release of complex particles composed of mucus, salts, and water [4]. Due to their low settling velocity, and to factors such as absolute humidity and droplet physical and chemical properties, these viruses can remain in the air for a long period of time [5,6]. 

Aside from being transmitted by air, viruses can attach to surfaces; their survival in environments outside of the host can increase the chance of transmission and future outbreaks [7,8,9]. Surfaces with a high virus survival rate are called fomites and can remain pathogenically active for between a few hours and several days. Strains of echovirus and poliovirus can retain their infectiousness on wood, glass, and fabric surfaces for 2 to 12 days, and sometimes for longer [10]. Additionally, human astroviruses can survive for more than 90 days on porous and non-porous materials [11]. In fact, physical, chemical, and biological factors can also influence the likelihood of virus survival [12,13]. 

The inactivation of viruses, which can be achieved through ultraviolet radiation, heat, free radical, and oxygen treatments, has been studied extensively [14,15,16]. The roles of copper and stainless steel have also been studied on model viruses [17], and it was shown that the likelihood of survival of aerosolized coronavirus is affected by metal surface type [18]. On the other hand, high-efficiency particulate air (HEPA) filters are commonly used to filter dust and bioaerosols [19]. In addition to high filter replacement costs, increased energy consumption (of heating, ventilation, and air conditioning (HVAC) systems), and high pressure drops [20], HEPA filters do not have cleaning or disinfecting properties or ways to inactivate viruses, which could result in their transmission through the air during filter replacement or disposal [21]. Respiratory syncytial virus (RSV) has been found in HVAC filters in daycare centers [22] and also in non-residential facilities where large crowds gather, such as subway systems, in which both genetic material of bacteria and viruses have been detected and could substantially increase the risk of outbreaks [23,24]. Also, viruses in ventilation systems are challenging to monitor via large-scale sampling. Ventilation systems could potentially be responsible for the spread of infectious diseases, such as legionella, influenza, smallpox, and even SARS-CoV [22,25,26]. Viruses can remain in the air for long periods and ventilation may play a role in outbreaks, including for the example of the recent SARS-CoV-2 pandemic [27]. For coronavirus, which presents a major threat worldwide, poorly ventilated indoor facilities [28] can promote its spread; however, hospital ventilation systems can prevent such transmission [29]. Currently, studies have been conducted on the air, surfaces environment, and contaminated personal protective equipment and there were traces of the coronavirus found in air outlet fans, although no investigations were conducted checking the infectivity of the virus [30]. While precautionary measures such as face masks and proper standard filter ventilation can protect a person from pathogens, these measures do not inactivate viruses on the surface of filters [31]. Based on the example outbreaks above, precautionary measures such as special filter technologies which have self-disinfecting properties could help stop transmissions and prevent outbreaks involving either airborne or aerosolized viruses.

This study conducted an investigation of the ability of Solid Oxygen-purifying (SOP) filters to self-clean or disinfect to prevent the spread of viable and inactivating aerosolized viruses, using MS2 bacteriophages as an example. SOP filters were highly enriched with what this study called oxygen-generating neutralizing (OGN) compounds, which are commonly used to convert air pollutants to oxygen. Another by-product of SOP filters is hydrogen peroxide (H_2_O_2_). H_2_O_2_ is unstable and can be converted into free radicals, which disable or kill bacteria and viruses [32,33,34]. Viruses that come into direct contact with SOP filters could be exposed to H_2_O_2_. 

This study tested SOP filters differing in density (g/m^2^), and compared them with HEPA filters and three layered commercial face masks (aerosolized viral loading), and soaked SOP with a high viral load of MS2 solutions at different time intervals to determine its effects and inactivation potential. 

## 2. Materials and Methods 

### 2.1. Manufacturing Solid Oxygen-Purifying (SOP) Filters

**Figure 1:**
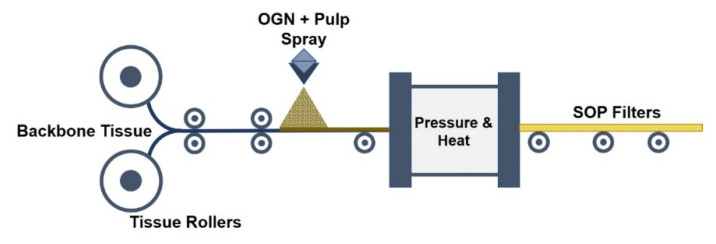
The air-laying process was used to make the solid oxygen-purifying (SOP) filters (conceptual diagram; not to scale). Tissues were sprayed with pulp/oxygen generating compounds and then exposed to pressure and heat to produce the Solid Oxygen-purifying (SOP) filters.

The Solid Oxygen-purifying filters used in this study were manufactured by Elstech., Inc. (Seoul, South Korea). The OGN compounds were alkali metals, such as calcium oxide, magnesium oxide, solid acid, and iron salts, which were ground into powder, mixed, and granulized with special binders. The enriched OGN material thus produced was used in an air-laying process. Two tissues were used as filter “backbones”. The tissues were sprayed with a mixture of 80 g of pulp and specific density of OGN material. Heat and pressure were used to complete the SOP filter manufacturing process (Figure 1). The SOP filters were patented and registered in the Korean Intellectual Property Office, South Korea.

**Figure 2:**
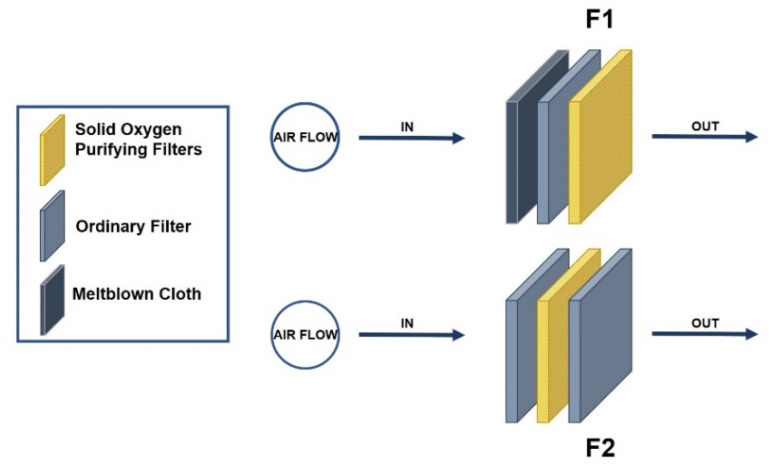
Different filter layer configurations. In configuration F1, the SOP (60 g/m^2^) is the final layer (covered by an ordinary filter and meltblown cloth). In configuration F2, the SOP filter is placed between two ordinary filters.

For the purposes of the experiment, Solid Oxygen-purifying filters differing in density were produced (15, 40, 60, or 100 g/m^2^). Other filters were added to create different configurations to compare their efficiency (combinations of meltblown (MB) fabric, ordinary, 60 g/m^2^ SOP filters), as illustrated in Figure 2.

### 2.2. Virus and Host Preparation

MS2 bacteriophage (Escherichia coli bacteriophage MS2, American Type Culture Collection [ATCC] 15597-B1; ATCC, Manassas, VA, USA) was used in this experiment, because this bacteriophage was used as a model virus in our previous and several studies [35,36,37]. The virus was prepared by adding high concentrations (approximately 100, 000 PFU/mL) of stock solution (100 μL) to 50 mL of sterilized deionized water. This solution was used as a viral aerosol.

The host (Escherichia coli, ATCC 15597) was pre-cultured prior to the experiment. A stock solution of E. coli (1000 μL) was prepared with 40 mL of sterilized Trypticase soy broth. This mixture was placed in a shaking incubator at 37 °C (speed of 150–200 rpm) and bacteria were cultured for 6–12 h. 

All of the stock solutions of virus and host were frozen (−75 °C) for the subsequent experiments, and a few stock solutions were incubated with bacteriophage to check viability. 

### 2.3. Experiment 1: Aerosolized MS2 Bacteriophage

**Figure 3:**
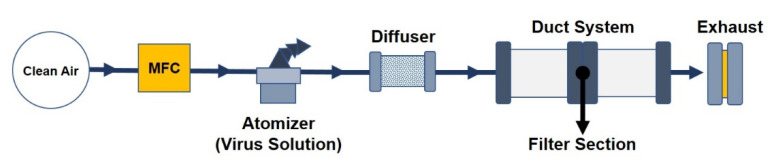
Aerosolized MS2 bacteriophage on filter samples. Clean air was produced from compressed air subject to high-efficiency particulate air (HEPA) filtering. It was then passed through a mass flow controller (MFC) and the atomizer.

The virus was aerosolized using a single jet atomizer (model 9302: TSI Inc., Shoreview, MN, USA) for 20 min, from pressure range of 180–200 kPa, and then passed through a diffuser and a 4 × 4 array of acrylic ducts, to which the filter samples were attached (Figure 3). HEPA filters (H14, 99.95%) were used as control filters because of the high survival rate of MS2 bacteriophage in contact with them [38]. In addition, commercial face masks with three layered MB filters (Anhui Xiangtianiun Medical Technology Co. LTD) were also used as another control for a comparison with SOP. 

All filter samples were cut into 1-cm-diameter pieces and then subjected to elution and dilution. The total contact time between SOP filters and virus particles (including control samples) was estimated to be about 30 min, because after the 20 min spraying process, we cut the filter into 1-cm-diameter pieces and submerged those pieces for 10 minutes in an elution solution. Also, the aerosolized virus was produced based on previous experiments [39].

### 2.4. Experiment 2: High Viral Load Exposure and Variable Exposure Time

To determine the efficiency of the SOP filters under a high viral load, 1-cm-diameter SOP filters were soaked with 1000 µL of pre-prepared MS2 bacteriophage. Liquid samples (1 µL) were then withdrawn for 1, 5, 15, 30 min or 60 min and diluted. The control samples for this experiment were a plain viral solution from the same source (with the same exposure times as those listed above but with no SOP filter added).

### 2.5. Calculations

**Figure 4:**
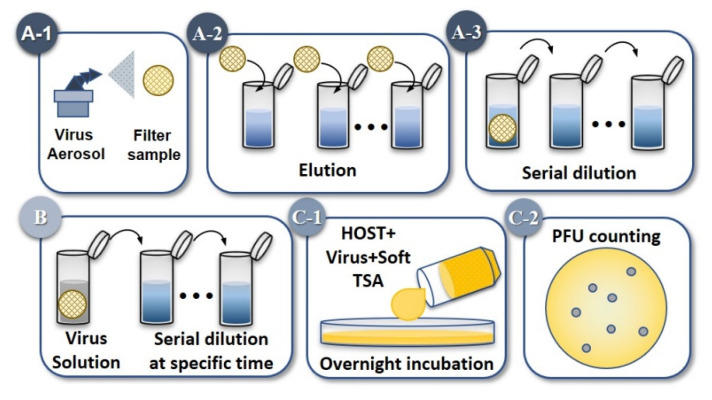
The experimental methods used in this study. (**A**) The aerosolization experiments involved spraying (**A-1**), elution (**A-2**), and serial dilution (**A-3**). (**B**) Microtube experiments with high viral load and serial dilution at specific time of sampling. About 100 µL of the liquid samples with different SOP were drawn and diluted. (**C**) Samples were then combined with a pre-cultured host. (**C-1**) PFU counting was done on the following day (**C-2**). In the figure, panels marked with A and B are being performed separately; those marked with C were performed after A and B are done.

Both experiments above involved plaque counting. Approximately 100 µL amounts of the control and SOP filter-treated diluted solutions were mixed with the pre-cultured host (300 µL), and added to 29 mL of soft agar (Trypticase soy agar, TSA). The mixture was placed on a 150-mm-diameter Petri dish and incubated overnight at 37 °C. The control and SOP filter-treated samples were compared in terms of the number of plaque-forming units per milliliter (PFU/mL); only samples with values of 100–1000 PFU/mL were considered in the comparison. The equation used to calculate the number of PFU/mL was as follows:

(1) $$C_{\left(\frac{PFU}{mL}\right)}=\frac{PFU}{\alpha }\times \frac{1}{d_{i}}$$

where *C* is the number of PFU/mL, PFU is the total plaque count (for every sample in the Petri dish), α is the volume of virus particles (1mL) added, and di is the dilution factor. The geometric mean of the plaque count was calculated and expressed logarithmically (base-10 logarithm [log10]). Differences in logarithmic values between control and SOP filter-treated samples were calculated as follows:

(2) $$Diff{\;}_{Log_{10\;C}}=Log_{10}\;\left(C_{Control}\right)-Log_{10}\;\left(C_{SOP}\right)$$

where *Diff Log_10_ C* is the difference between the control and SOP filter-treated sample plaque counts (logarithmic values). The virus inactivation efficiency of the SOP filter was calculated as follows:

(3) IE(%)={1−(CSOPCcontrol)}×100

where *IE*(%) is the inactivation efficiency, *C_SOP_* is the number of plaque-forming units per milliliter of sample treated with Solid Oxygen-purifying filters, and *C_control_* represents the control samples. The sample preparation and PFU calculations were based on a previous study [17,39] Furthermore, the experimental methods of this study are summarized in Figure 4. In both experiments, sampling was performed in triplicate at room temperature (25 °C).

## 3. Results

### 3.1. MS2 Bacteriophage Inactivation Efficiency on Filters Configurations

Inactivation efficiency was relatively high in the aerosolization experiments for most of the Solid Oxygen-purifying filters (93.5 ± 1.06%, 94.7 ± 0.18%, and 99.4 ± 2.17% for the 40, 60 and 100 g/m^2^ filters, respectively). A lower inactivation efficiency of 52.6 ± 1.15% was calculated for the 15 g/m^2^ filter, suggesting that the density of the SOP filter was directly proportional to its efficiency. Furthermore, the 15 g/m^2^ SOP filter had a lower log10 PFU/mL value, of 0.53, than the 40, 60, and 100 g/m^2^ filters (1.32, 1.59, and 2.32 log_10_ PFU/mL, respectively).

Comparing the two different filter layer configurations, F1 (in which MB cloth was first exposed to virus particles, followed by the ordinary and SOP filters) had a significantly lower log_10_ PFU/mL value for the difference between the control and SOP filter-treated sample plaque counts (0.57) than F2 (in which the Solid Oxygen-purifying filter was sandwiched between two ordinary filters; log_10_ PFU/mL = 1.36). In Table 1, the F1 and F2 configurations also differed in terms of virus inactivation efficiency (81.8 ± 1.26% and 94.6 ± 2.08%, respectively). 

### 3.2. Effects of Exposure Time (High Viral Load)

**Figure 5:**
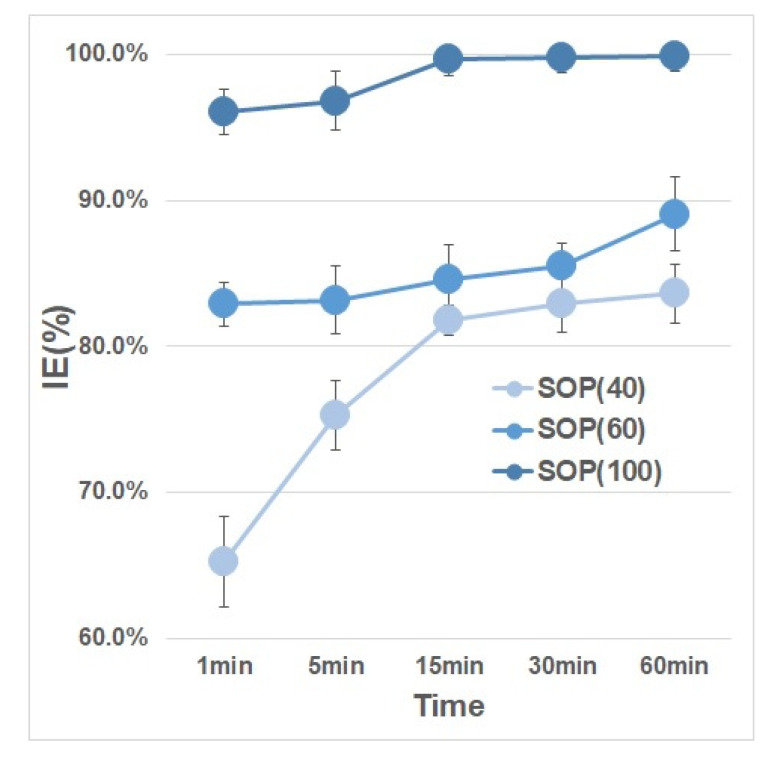
Comparison of the inactivation efficiency (IE) of the various SOP filters by exposure time.

To determine the effect of exposure time on MS2 bacteriophage inactivation, high concentrations using 1000 µL of stock solution MS2 bacteriophage were employed. As shown in Figure 5, lower-density SOP filters (40 and 60 g/m^2^) had lower inactivation efficiencies of 65.2 ± 3.10% and 82.9 ± 1.50%, respectively, after 1 min. The 40 g/m2 SOP filter had inactivation efficiencies of 75.3 ± 2.36%, 81.8 ± 1.02%, 83.0 ± 2.03%, and 83.6 ± 2.03% after 5, 15, 30, and 60 min, respectively; the respective values for the 60 g/m^2^ filter were 83.2 ± 2.30%, 84.6 ± 2.36%, 85.5 ± 1.59%, and 89.1 ± 2.56%. Only the 100 g/m^2^ SOP filter had inactivation efficiencies greater than 95%; these were 96.1 ± 1.56%, 96.8 ± 1.56%, 99.7 ± 1.20%, 99.8 ± 1.10%, and 99.9 ± 1.03% after 1, 5, 15, 30, and 60 min, respectively. These results showed that a longer exposure time increased the MS2 bacteriophage inactivation efficiency. The 15 g/m^2^ Solid Oxygen-purifying filters were not included in these experiments due to their high solubility in water.

### 3.3. Comparison of Different Controls

For these experiments, both commercial surgical mask and HEPA filters were used as controls to compare the inactivation properties of SOP filters. The aerosolized experiments involved spraying and elution times of 20 and 10 min, respectively (30 min total contact time between SOP filters and virus particles) so results at 30 min interval were compared in this section. This also includes the results on the high viral load experiments (control samples are high virus solutions without SOP) after a 30 min time interval.

The inactivation efficiency in the aerosol experiments, in which the viral loads were lower (due to loss during spraying) was up to 93.5 ± 1.15%, compared with up to 83.0 ± 2.10% for the 40 g/m^2^ Solid Oxygen-purifying filter under the high viral load condition. The 60 g/m^2^ SOP filter had an inactivation efficiency of 94.7 ± 1.06%, compared with 85.5 ± 1.25% under the high viral load condition. Only the 100 g/m2 SOP filter showed a similar inactivation efficiency performance between the aerosol and high viral load experiments (99.4 ± 0.18% and 99.8 ± 0.16%, respectively). 

**Figure 6:**
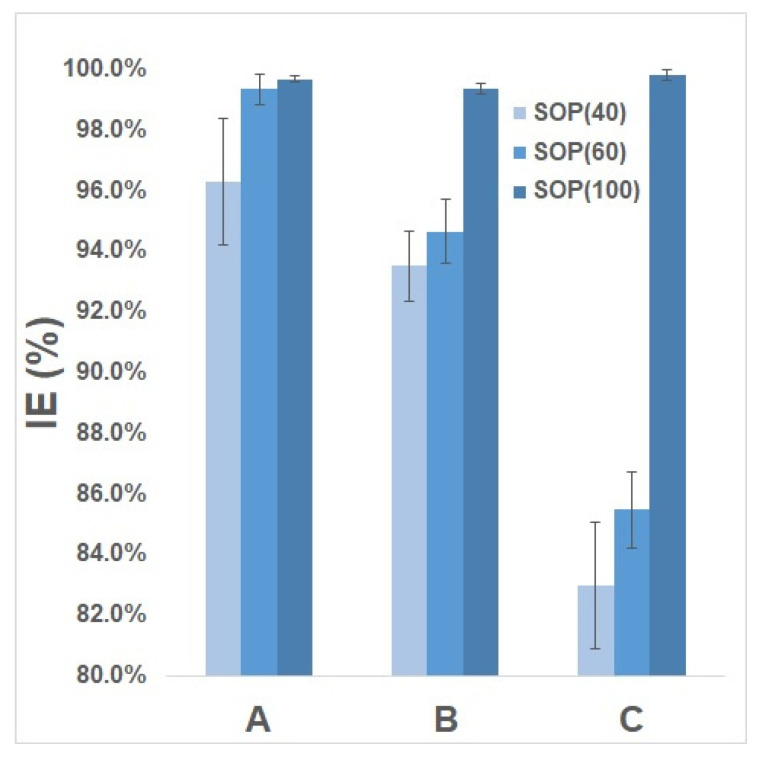
Comparison of inactivation efficiency (IE) of SOP (solid oxygen-purifying) between the commercially available surgical face mask (**A**), HEPA filters (**B**) and soaking with high viral loading (**C**). Both A and B used aerosol transmissions which was attached on the surface of face mask, HEPA and SOP Filters, while C used a microtube filled with (1000 µL) MS2 bacteriophages, where a 1 × 1 cm diameter of SOP filter was soaked. Only the data with the same time interval (30 minute) were being compared.

Also, the experiment determined the self-disinfecting potential of SOP compared with a commercially available three-filter layered surgical mask. As the results show, the inactivation of MS2 was higher compared to the commercial facial mask conducted with this study, which had 96.3 ± 2.1%, 99.3 ± 0.5%, and 99.7 ± 0.1%, with respect to a SOP density of −40, −60 and −100 g/m^2^. The increase in inactivation was due to the lower MS2 attachments on these commercial face mask during aerosolized experiments, which meant that HEPA filters could filter these viral aerosols more effectively. These results are presented in Figure 6.

## 4. Discussion

The major purpose of HEPA filters is to prevent the release of dust particulates and a range of microorganisms into the air. Although these filters are common in air filtration systems, they cannot inactivate bacteria and viruses. As a report showed that MS2 bacteriophage, could survive on the surfaces of HEPA filters for up to 7 days [38]. Additionally, there are no standards or specifications regarding the use of HEPA filters in some countries, such as South Korea [40].

This study used Solid Oxygen-purifying filters with a high content of OGN compounds, which can produce H_2_O_2_ as a by-product. The filters, which are as effective as disinfectant-type SOP filters, were made from an alkali metal oxide ([M]O_2_), which converts to oxygen upon coming into contact with an air pollutant ([A]O_2_), CO_2_, SO_x_, or NO_x_ (Equation (4)). The virus inactivation mechanism of these filters is based on H_2_O_2_ (Equation (5)), which can be converted by catalase into oxygen (Equation (6)). H_2_O_2_ has a very unstable bond that can be broken at any time; the free radicals thus formed, such as the hydroxyl radical (·OH), tend to have sterilizing properties. These compounds can only serve as a highly effective disinfectant if the contact time is sufficient time and the concentration is between 7.5% and 30% [32,33,41,42,43,44].

(4) $$\left[A\right]O_{2\;}+\left[M\right]O_{2}\;\to \;M_{x}\left[A\right]O_{y}+\;O_{2}$$

(5) $$\left[A\right]O_{2}\;+\left[M\right]O_{2}\;+\;H_{2}O\;\to \;M_{x}\left[A\right]O_{y}+\;H_{2}O_{2}$$

(6) $$H_{2}O_{2}+cat\;\to \;H_{2}O+\frac{1}{2}O_{2}$$

(7) $$2H_{2}O_{2}\to OH•+HOO•+H_{2}O$$

Based on the results of this study, SOP filters show promise for inactivating aerosolized viruses. High inactivation efficiencies of up to 99.8% were obtained in the experiments. These filters could be used in various applications, such as in HVAC systems (added to the standard HEPA filter systems) and face masks. Solid Oxygen-purifying filters could be used in commercially available face masks because of their efficacy in converting pollutant species such as CO_2_, SO_x_, and NO_x_, and even volatile organic compounds, into oxygen. Technologies for use in face masks to filter airborne diseases are currently in high demand. 

Surgical face masks and N95 respirators have shown high efficiency in filtering pathogens such as influenza and rhinoviruses [45], whereas homemade face masks only provide about half the protection of surgical masks [46]. The need to wear a protective mask during global outbreaks, such as the coronavirus pandemic, especially in hospitals where the risk of transmission of airborne pathogens is high, has been suggested previously. For example, given the possibility of pre-symptomatic transmission, healthy people may benefit from masks [31]. Additionally, wearing a face mask is likely to greatly reduce the spread of the virus [47]. However, even though masks can prevent transmission, current filters do not reduce or eliminate pathogens; viruses remaining on the surface of a mask could still pose a threat. SOP filters could be a solution to this problem because they have the potential to inactivate airborne and aerosolized viruses. Another drawback of face masks is that their efficiency is lowered due to long usage. Solid oxygen-purifying filters have the potential to self-disinfect and could potentially last longer. Due to some limitations, this study only performed assessments on MS2 bacteriophages; therefore, further studies are needed on SOP filters such as its shelf-life, recyclability, and effectivity on different types of bacteria and viruses that are pathogenic in nature. 

As shown by the results in this study, the effectiveness of Solid Oxygen-purifying filters is not only based on their density, but also on the direct contact of the filter with virus particles. For the filter configurations used in this study, the filters were designed based on a real-life setting where the SOP filter was inserted between the mouth and the face mask. MB fabrics were used because they are manufactured on a large scale in South Korea, such that the experimental data have real-world validity. These nonwoven fabrics are produced directly from polymers or resins through a melt-blowing process using high-velocity air and other forces that promote attenuation of filaments. The filtering efficiency of MB fabrics was previously shown to be superior to that of glass fiber HEPA filters [40,48]. In this study, MB fabric in the F1 filter configuration was directly exposed to the aerosolized MS2; an ordinary filter and the SOP filter were placed behind the MB fabric. In the F2 configuration, the SOP filter was sandwiched between two ordinary filters. The relatively low inactivation efficiency of the F1 configuration may have been due to the MB fabric only filtering MS2 bacteriophage during spraying, or to the virus particles only being attached to (i.e., trapped on) the surface of the MB. Thus, it may have been difficult for the virus to pass through the MB and reach the SOP filter. In contrast, in the F2 configuration, the ordinary filters allowed the virus to pass through easily and reach the SOP filter. This suggests that Solid Oxygen-purifying filters could be effective for inactivating viruses originating from the mouth of the individual wearing the mask, thus preventing viruses from spreading. Also, commercial surgical masks were compared and it was found that SOP could self-disinfect more than an ordinary mask. Also the company that provided the SOP filters was manufacturing these materials (at lower OGN density) as add-ons for ordinary facial masks, which passed tests on the safety of skin contact based on local standards of different testing research in South Korea [49].

Filter density was also key for effective virus inactivation; in fact, filter density was directly proportional to the degree of MS2 bacteriophage reduction. However, solid residues and OGN material detached from the tissue backbone of the filter, thus creating SOP dust. This could have increased exposure during elution, when OGN compounds began to precipitate on the sample micro tubes. Decreasing the amount of OGN compounds also compromised the integrity of the SOP filters; in the case of the 15 g/m^2^ SOP filter, the compounds dissolved in the distilled water under high viral loading, and were difficult to retrieve. Based on this study, the aerosolized viruses could be more than 90% inactivated with densities of 40 and 60g/m^2^, which could be a standard on SOP-filters. For the excessive dust of SOP on higher density, increasing the tissue backbone could reduce this dust but airflow might also be compromised.

In addition, these filters could be attached onto an air ventilation system and due to its properties of self-disinfecting, the system could then promote better air quality. There are other methods to address the issue on bacterial and viral aerosols; some of these technologies include non-thermal plasma barriers [50] and ultraviolet light [51]. These technologies could reduce the spread of airborne viruses transmitted in the air, yet their drawbacks are their reduced operation, increased power consumption, and higher maintenance and cost requirements. Solid Oxygen-purifying filters might help these technologies to provide better reduction of bacterial and viral particles. Moreover, technology on making SOP filters is readily available, commercialized, and ready-to-use, which could help reduce the spread of outbreaks.

HVAC should be considered for producing cleaner air by not just filtering dust but also by developing and installing low cost filters such as the SOP, which could inactivate microorganisms to prevent the future spread of deadly diseases.

## 5. Conclusions

Solid Oxygen-purifying filters inactivated MS2 bacteriophage, thereby indicating their potential for several different applications. SOP filters could be used in HVAC systems and face masks to protect against certain types of infections. The SOP filter configurations used in this study shed light on the filter placement needed for highly efficient virus inactivation. The results of other studies pertaining to the reduction of viral aerosols could be combined with our results to improve filter performance. Furthermore, the self-disinfecting properties of a SOP filter could be studied more in order to have a wide range of insights and understand their many applications. 

## Figures and Tables

**Table 1:** Inactivation Efficiencies (%) and Logarithmic difference of aerosolized MS2 bacteriophages in each filter treatment.

Filter Type	Density (g/m^2^)	*IE (%)*	*Diff (_Log10PFU/mL_)*
SOP	15	52.6 ± 1.15%	0.53
	40	93.5 ± 1.06%	1.32
	60	94.7 ± 0.18%	1.59
	100	99.4 ± 2.17%	2.32
F1 – config.	60	81.8 ± 1.26%	0.57
F2 – config.		94.6 ± 2.08%	1.36
